# Human–nature interactions and the consequences and drivers of provisioning wildlife

**DOI:** 10.1098/rstb.2017.0092

**Published:** 2018-03-12

**Authors:** Daniel T. C. Cox, Kevin J. Gaston

**Affiliations:** Environment and Sustainability Institute, University of Exeter, Penryn TR10 9FE, UK

**Keywords:** bird feeding, extinction of experience, resource provisioning, urbanization, urban wildlife, wildlife gardening

## Abstract

Many human populations are undergoing an extinction of experience, with a progressive decline in interactions with nature. This is a consequence both of a loss of opportunity for, and orientation towards, such experiences. The trend is of concern in part because interactions with nature can be good for human health and wellbeing. One potential means of redressing these losses is through the intentional provision of resources to increase wildlife populations in close proximity to people, thereby increasing the potential for positive human–nature experiences, and thence the array of benefits that can result. In this paper, we review the evidence that these resource subsidies have such a cascade of effects. In some Westernized countries, the scale of provision is extraordinarily high, and doubtless leads to both positive and negative impacts for wildlife. In turn, these impacts often lead to more frequent, reliable and closer human–nature interactions, with a greater variety of species. The consequences for human wellbeing remain poorly understood, although benefits documented in the context of human–nature interactions more broadly seem likely to apply. There are also some important feedback loops that need to be better characterized if resource provisioning is to contribute effectively towards averting the extinction of experience.

This article is part of the theme issue ‘Anthropogenic resource subsidies and host–parasite dynamics in wildlife’.

## 1. Introduction

It is widely accepted that many human populations are undergoing a progressive extinction of experience of nature [1–3]. Particularly in more developed countries and regions, over recent decades regular daily contact of people with nature has been in persistent decline. This is especially marked in children, with often increasingly stark differences between the nature experiences (e.g. visiting natural areas, watching wild animals, climbing trees) of the present generation when compared with those of their parents, grandparents and great-grandparents [4].

The extinction of experience is arguably an inevitable consequence of the growing urbanization of the human population, which in 2007 passed the point at which across the world one in every two people lived in a town or city [5]. Urban lifestyles tend to reduce the likelihood of inevitable daily interactions with nature, and increase the necessity of intentionality (e.g. in visiting greenspaces) to experience such interactions. The extinction of experience has also been fuelled by global and regional losses of natural habitat and biodiversity (e.g. [6,7]), and the growth of sedentary pastimes such as watching television, and engaging with the Internet and social media (e.g. [8,9]). Indeed, for increasing numbers of people ‘nature’ has become something primarily accessed through filters of parents, peers and/or the media; there is virtually no personal interaction involved.

The consequences of the extinction of experience are increasingly thought to be profound. First, there is compelling evidence that the loss of nature interactions has negative impacts on multiple dimensions of human health and wellbeing [10,11]. These include effects on physical health [12,13], mental health [14,15] and social wellbeing [16]. Second, there is evidence that reduced nature experiences can lead to people having less affinity to and interest in nature (e.g. [17,18]), placing less value on nature (e.g. [3,18]) and being less likely to participate in pro-environmental behaviours (e.g. [19,20]).

**Figure 1.**
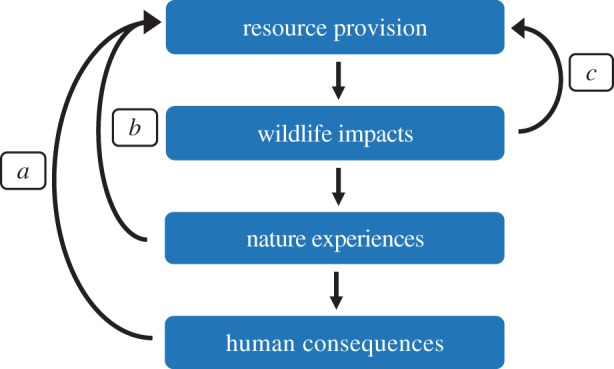
The potential links between urban resource provisioning, wildlife, and human–nature interactions and their consequences. The human consequences can lead to feedback loops in which the consequences accelerate or decelerate further resource provisioning: feedback *a*, health and wellbeing benefits from experiencing wildlife increase resource provision; feedback *b*, anticipation of wildlife experiences as yet unrealized increases resource provision; feedback *c*, a concern for wildlife welfare without experiencing wildlife increases resource provision. Note that the schematic diagram does not represent all potential factors and processes. (Online version in colour.)

One conscious or subconscious response to the extinction of experience might be the intentional provision of resources to wildlife, as people seek to enable or to increase daily nature experiences. Indeed, such provisioning of resources is conducted on a massive scale in some regions. In this paper, we examine the links between such resource provisioning, impacts on wildlife, and the impacts on human–nature experiences and their consequences (figure 1). We focus on the purposeful provision of resources by the general public for wildlife. We do not address intentional provisioning for scientific research, conservation, management or tourism (e.g. see [21]) or the unintentional provision, such as from human food waste, that can occur, often at scale, in and around towns and cities. We also focus almost exclusively on urban areas, because these are environments in which people not only have reduced exposure to nature and so the extinction of experience is particularly prevalent, but also are where levels of wildlife provisioning tend to be greatest (e.g. [22]). These are also areas in which high densities of people and some animals live together, and so where the benefits and costs of resource provisioning are most starkly revealed. In the main, our examples are drawn from Western societies, which at present are the foci of documented resource provision activities. The extent to which this is a cultural and/or economic constraint remains to be determined.

## 2. Resource provision

The intentional provision of resources for wildlife, often under the banners of ‘urban greening’ and ‘wildlife gardening’, can include food, water, general habitat and breeding, roosting and wintering sites [23–25]. The most common location in which people provide such resources is within their domestic gardens [24]. These land parcels comprise a substantial proportion of the mosaic of land use in urban areas and are often in aggregate the largest component of greenspace. For example, domestic gardens constitute 16% of the area of Stockholm, Sweden [26], 36% of Dunedin, New Zealand [27], and 19–27% of cities in the UK [28]. In the UK, an estimated 87% of households have access to a private garden, varying from a few square metres to several hectares [24]. Although small in themselves, scaled-up to the national level the resources they provide are significant, such that in the UK it has been estimated that domestic gardens contain 2.5–3.5 million freshwater ponds and 28.7 million trees (just under a quarter of all trees occurring outside of woodlands; [24]). Resource provisioning includes that of both native and non-native plants, provided not only for the aesthetic appeal, but also to attract butterflies, bees (and other pollinators) and birds (e.g. [29,30]). The extent of these activities within individual gardens can vary enormously, from leaving a nettle *Urtica dioica* patch untouched or the installation of an artificial home for invertebrates, to full-scale management for biodiversity [31,32].

**Figure 2.**
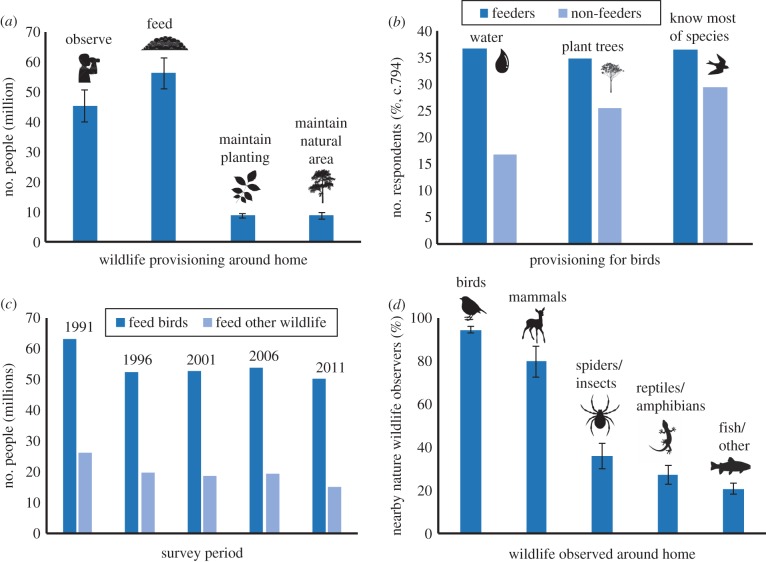
Empirical evidence demonstrating levels of resource provisioning around the home. (*a*) Feeding wildlife is the most common nature interaction in the USA [37]; (*b*) in New Zealand people who feed birds are more likely to engage in other resource provisioning activities [38]; (*c*) trends of wildlife feeding around the home in the USA [37]; and (*d*) birds are the most observed wildlife around the home in the USA [37]. (Online version in colour.)

The provision of supplementary resources for birds is undoubtedly the most popular form of resource provisioning, driving a multi-billion dollar global industry [33,34]. The level and range of supplementary feeding can be astounding [35]. In the UK, there are approximately 12.6 million (48%) households providing food for birds [24], that is an average feeder density across the UK of 100 per km^2^ and about 200 per km^2^ in one city of half a million people [36]. Or, put another way, equating to one bird feeder for every nine potentially feeder-using birds in the UK [24], and providing enough resource to feed almost three times the breeding populations of 10 feeder-using songbird species [35]. Likewise, annually in the USA an estimated 54.3 million (73%) households provide 500 000 tonnes of food (figure 2*a*), sufficient to feed 300 million chickadees *Poecile* spp*.* if they fed on nothing else [39]. Similarly, there are a minimum of 4.7 million nest-boxes within gardens in the UK, at least one nest-box for every six breeding pairs of cavity nesting birds in the country [24]. Further, people who feed birds or put up nest-boxes are also more likely to provide other resources for birds, such as through planting trees or providing water (figure 2*b*). Songbirds are not the only beneficiaries, with other examples including the provision of meat for red kites (*Milvus milvus*) in the UK [40], and for butcherbirds and magpies in Australia [41], hand-feeding of bread to American white ibis (*Eudocimus albus* [42]) or fruit for cassowaries (*Casuarius casuarius johnsonii*) in northeast Australia [43].

Usually more opportunistically than with garden bird feeding, people also provide food resources for mammals in urban areas. In the USA, the scale of backyard feeding of racoons, squirrels, skunks, bears, coyote and deer either intentionally, or indirectly via bird feeders, is difficult to quantify, because unlike supplementary food for birds there is no equivalent measurable product for mammals. However, by surveying local residents it is possible to estimate the energetic quantity supplied [44]. There is evidence that in some urban areas provisioning may be significant, with the opportunistic feeding of mule deer, O*docoileus hemionus*, and white-tailed deer, *O. virginianus* [21], and bears [45] by locals being cited as a factor contributing to the subsequent culling of animals [46]. In Western Europe, nocturnal urban mammals, such as hedgehogs, *Erinaceus europaeus* [47], and foxes, *Vulpes vulpes* [44], are the focus of subsidy provisioning, with 92% of 253 dead hedgehogs in Finland being found with human-fed foods in their stomachs (especially fish and milk; [48]).

## 3. Wildlife impacts

As resource provisioning continues to grow in popularity, it is increasingly important to understand the ecological impacts that these huge quantities of additional material have on both target and non-target wildlife populations. The provision of wildlife-friendly habitats and/or food-bearing plants has been associated with attracting a wider community of invertebrate species [30], birds (e.g. [49]) and an increase in the proportion of gardens used frequently by hedgehogs and mice (e.g. [50]). Indeed, small gardens managed for wildlife can be as rich in terms of biodiversity as large gardens, overriding the effects of size and location within the urban matrix [23,28,51].

Many of the songbirds that are the target of resource subsidies are already relatively common [52], with provisioning being associated with their increased local abundance and distribution [53,54]. Given that these species are in some cases also in major regional decline (e.g. [7]), this begs the question as to the extent to which their populations are effectively being ‘propped up’ by such provisioning. The impacts of resource subsidies can also influence the local abundances, particularly in urban areas, of species as diverse as red kites, *Milvus milvus* [55], hummingbirds (e.g. [56]), blackcaps, *Sylvia atricapilla* (e.g. [57]), and macaques [58]. It is currently unclear whether, and under what circumstances, the higher abundances of these species are associated with real increases in population abundances, or whether they are a result of displacement effects whereby resource provisioning drives local immigration [59].

The provision of easily accessible resource subsidies has been associated with positive outcomes for some target bird species, including but not limited to, increased adult overwinter survival [56,60,61], earlier lay dates and increased egg and clutch sizes [39]. Its effects on bird productivity are variable, and overwinter feeding has been found both to increase [39] and reduce [62,63] breeding success in subsequent seasons. Similarly, experiments during the breeding period have found mixed results, with evidence for both increases (e.g. [64]) and reductions [65] in productivity. Birds are not the only recipients potentially to benefit from resource provisioning, with around a third as many people in the US feeding other wildlife (figure 2*c*). The intentional feeding of Eastern chipmunks, *Tamias striatus*, allows them to maintain their activity levels from spring until autumn, without the summer lull that is seen in their rural counterparts [66]. High densities of food allow some urban mammal species to reduce their home range size compared to their rural counterparts (e.g. racoon, *Procyon lotor* [67]; Florida Key deer, *Odocoileus virginianus clavium* [68]) and has been attributed to buffering urban populations of species against severe weather events (e.g. Hanuman langurs, *Semnopitheaus entellus*, in India [69]).

As well as conveying benefits, supplementary feeding can also have negative consequences for target species of wildlife. Most prominent, is the increased risk of pathogen transmission through increased contact rates between hosts, and pathogens accumulating at feeders and in the surrounding environment (reviewed by [70,71]). Indeed, pathogen transmission in house finches (*Haemorhous mexicanus*) has been found to be significantly higher in birds in areas with high densities of bird feeders [72]. Feeder-related disease transmission is thought to have contributed towards the rapid population declines of once common species (e.g. *Trichomonosis gallinae* in greenfinches, *Carduelis chloris*; [73]). There is also evidence of an increased risk from supplementary feeding of local songbird nest predation [74], delays in the start of dawn singing [75] and changing predator–prey dynamics [76], while the provision of inappropriate foods can result in poor welfare (e.g. magpies and butcherbirds [77], cassowary [43]). It would be surprising if the provision of resource subsidies did not also impact on some other species. For example, the provision of mostly bread through garden bird feeding in New Zealand benefits introduced species (which are predominantly granivores or omnivores) at the expense of native ones (which are predominantly insectivores and nectarivores; [59]). Further, resource subsidies can result in decreases in local abundances of some non-target species, such as ground beetles that fall prey to ground-foraging birds attracted to the feeders [78], and increases in others, such as introduced grey squirrels, *Sciurus carolinensis* [79], and ring-necked parakeets, *Psittacula karameri*, in the UK [80]. Grey squirrels, for example, have been shown negatively to impact on resource acquisition by songbirds by over 90% [79], likely because of strong interference competition between songbirds and this dominant aggressive species monopolising resources [39].

Opportunistic backyard feeding of large, potentially dangerous animals has been associated with few benefits, but numerous costs for wildlife. Feeding inevitably leads to changes in behaviour and ecology of these species, including increased aggression and frequency of conflict behaviours, which can often lead to the need for the removal of problem individuals (e.g. southern cassowary in Australia [43], deer and bears in USA [21]). The hand feeding of primates appears to be common across cultures and countries, leading to behavioural changes, particularly increases in aggression and enhanced risk of road traffic fatalities (e.g. long-tailed macaques, *Macaca fascicularis*, in Singapore [81] and Hanuman langurs, *Semnopitheaus entellis*, in India [82]).

## 4. Nature experiences

**Figure 3.**
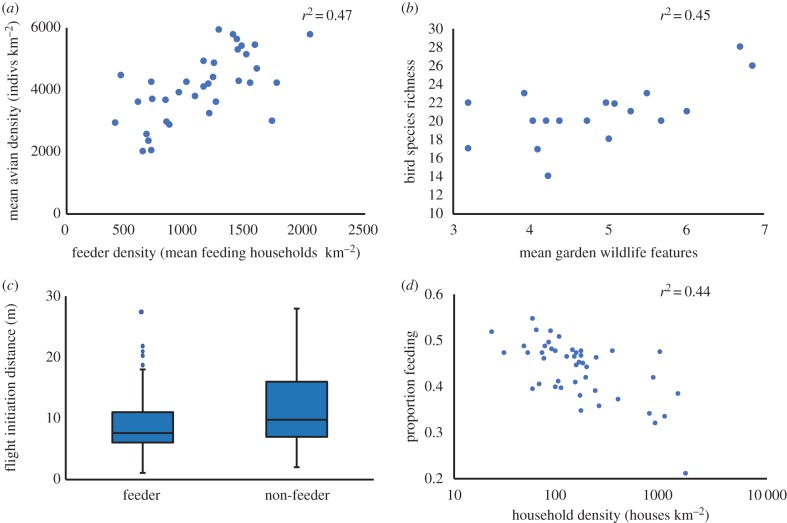
Changing wildlife experiences. (*a*) Increased bird abundance with feeder density in Sheffield, UK [54]; (*b*) increased bird species richness with the number of wildlife-friendly features in gardens in Leeds, UK [25]; (*c*) flight initiation distances of different bird species decrease in the presence of bird feeders in urban areas [83]; and (*d*) the proportion of people feeding birds decreases in compact urban designs [84]. (Online version in colour.)

Inevitably, such a wide range of ecological impacts resulting from resource provisioning is bound to influence people's experiences of the wildlife around them. Fundamentally, the increased density of resources in a garden, or group of gardens, will increase the flow of target (and some non-target) species into these areas (figure 3*a*). The resulting increased abundances are then likely to increase the frequency and duration of human–wildlife interactions. Further, the increased density of subsidies is also likely to increase the number of individuals and species that are seen at any one time, especially when a variety of resources are provided (figure 3*b*). Resources are usually placed in visible locations, thereby increasing the reliability of sightings, particularly of rarer species or ones with more cryptic behaviour (e.g. nocturnal ones such as hedgehogs [47]). Resource subsidies can also be associated with behavioural shifts, including increased boldness and neophilia [85], reduced flight initiation distances (figure 3*c*), facilitating an ability to discover new food sources more quickly [86] and allowing overwintering of some otherwise migratory species (e.g. [57]). In sum, these changes can allow people to view animals at a closer proximity, more reliably, for longer and throughout the year.

People tend to feed wildlife that they experience daily, with birds being the most commonly encountered (at least in terms of that wildlife which is recognized) around the home (figure 2*d*). In many urban areas introduced species outcompete native ones [38], therefore changing people's experiences of local bird communities. Indeed, increasing numbers of children [87] and adults [88] can now more easily recognize introduced as opposed to native species. A potential consequence of this desire to interact with everyday nature is the provision of subsidies appropriate for introduced but not native species [38], thereby exacerbating the problem. It is currently unclear what effect, if any, this shifting species baseline has on the extinction of experience or the conservation of native species (discussed in [89]).

One consequence of the extinction of experience is not only a reduced exposure to nature, but also that people may fail to note the nature that they do encounter, through a lack of familiarity and knowledge [3]. Resource provisioning means that people expect to see wildlife in, on and around these subsidies, and so are more likely to experience the wildlife that is there. However, increasing local abundances is not necessarily sufficient to maintain or increase people's daily connection to nature. As the global human population becomes increasingly urbanized, a critical question is how can we design urban areas not only to foster positive daily experiences of nature, but also people's desire to seek out these experiences [18]. Those communities living in compact urban landscape designs have reduced associated wildlife populations, with households being less likely actively or passively to engage with nearby nature [90], or to provide food for birds (figure 3*d*). Therefore, communities that are already deprived across multiple dimensions of health further lose access to these experiences. More sprawling urban landscape designs, with increased numbers of neighbourhood greenspaces promote population sizes of wildlife species for multiple taxa [91], facilitating more frequent daily wildlife experiences [91,92].

## 5. Human consequences

As resource subsidies change people's everyday experiences of wildlife, so too are they likely to influence the health and wellbeing outcomes that people receive from exposure to nature. Evidence suggests that an increase in the intensity of exposure is associated with improved health outcomes, with health gains increasing with both the quantity and quality of the natural elements that are encountered [93]. Resource subsidies can be seen positively to influence exposure quantity through an increased abundance of target and non-target species, and exposure quality through attracting an increased number of species.

**Figure 4.**
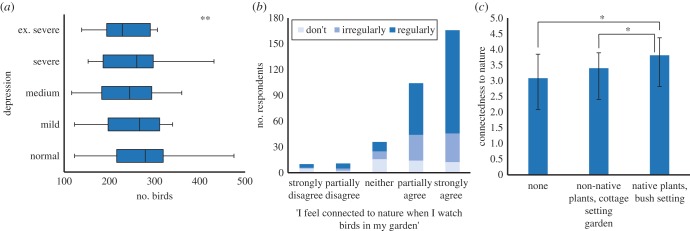
Variation in health and wellbeing benefits associated with nature and nature experiences. (*a*) A lower population prevalence of depression was associated with higher afternoon abundances of neighbourhood birds in the UK [15]; (*b*) in the UK people who fed birds regularly felt more connected to nature when they watched birds in their garden [95]; and (*c*) people who provided resources in Australia had a higher connectedness to nature, than those with ecologically poor gardens [97,98]. (Online version in colour.)

Increasing exposure quantity through an increase in the abundance of resources provided has been positively associated with increases in psychological benefits, such as feelings of pleasure [33,94], and of being relaxed and connected to nature [95]. A greater number of birds means more birdsong, which contributes towards perceived attention restoration and stress reduction [96]. Cox *et al*. [15] found positive associations between people's mental health and the numbers of birds in their neighbourhood in the afternoon (when people are more active), but not the numbers of birds in the morning (when birds are more active; figure 4*a*). They concluded that mental health benefits from neighbourhood nature were likely associated with the birds that people encounter, as opposed to their intrinsic abundance. A logical next step is that a feeder, which attracts birds to where they can more easily be seen by people, has the potential to provide a focal point that might contribute to the prevention and treatment of poor mental health.

Increasing exposure quality through an increase in the variety of resource subsidies has been positively associated with people's preferences for urban gardens [99], birds at feeders [100] and bird song [101]. However, the paradox is that most people are usually unable to appreciate richness by themselves, and so instead may gain benefits from perceived richness (discussed in [102]). A bird feeder can be seen to close the gap between actual and perceived richness, even for people with limited knowledge about the species, because it allows the viewing of multiple species within a short timeframe.

Resource subsidies provide easier access to daily interactions with wildlife around the home, which has been associated with a greater empathy for, and understanding of, the natural world [34,92,103]. Indeed, an increased connection to nature is associated with greener more diverse gardens [90], and with people being more likely to provide resources for wildlife (figure 4*b*). In Australia, those people with an increased orientation towards nature were more likely than those with a low nature orientation to have native plants in their garden and live in a bush setting, and be less likely to have ecologically poor gardens (figure 4*c*). People who fed birds regularly tended to know the names of more garden species, to consider these species to be more likeable [38,100], and to feel a greater connection to nature when they watched birds in the garden [95].

Although the opportunistic feeding of large dangerous animals doubtless provides those doing it with immediate feelings of connection to nature and wellbeing, the inevitable habituation of fed individuals can, and often does, lead to longer term negative consequences for human health and wellbeing. Feeding has been associated with an increase in the number of human–wildlife conflicts (see [21]). At the worst, attacks can lead to serious injury and death (e.g. by cassowaries [43] or dingoes [104]), while other issues include noise, mess, the destruction of property and attacks on domestic animals (e.g. [105]). The costs of conflict resulting from resource provisioning are difficult to quantify because most conflict is relatively minor, and it is difficult to assess costs such as diminished psychological wellbeing and disruption of livelihoods [106].

## 6. Feedbacks

To this point, we have presented a rather linear sequence of events in which resource provisioning affects wildlife, which in turn affects human nature experiences, which has consequences for the recipients of those experiences. However, the situation is doubtless more complex. There are, of course, likely to be a number of feedback loops (figure 1). Most obviously, if people gain health and wellbeing benefits from resource provisioning [107,108], and from seeing wildlife in their garden (e.g. [34,95]), then they may be more likely to persist with providing resources where these encourage more wildlife [25,44,109]; figure 1, feedback *a*).

Further, resource provisioning may be an expression of an orientation towards nature. Nature orientated people may be responding to a reduced opportunity to regularly experience nature, by attempting to manipulate local wildlife populations, thereby allowing closer, more meaningful interactions. Cox and Gaston [95] found that people who regularly fed birds were willing to do so even if there were none currently in the garden, although this willingness decreased in people who fed birds irregularly or did not feed birds. This suggests a feedback loop whereby those people who are orientated towards nature may provide resources because they anticipate positive human consequences (figure 1, feedback *b*). Conversely, a failure to use resources by wildlife, for example due to a decline in the local wildlife population [44], may decrease the desire to continue with provision by people with a low nature orientation.

Undoubtedly, many people provide resources due to motivations grounded in species conservation and welfare (e.g. [95,110]), such as ‘helping’ songbirds during periods of harsh winter weather [34]. This is despite during these periods daylight hours being shorter, with people spending less time in their gardens so arguably there being less likelihood of experiencing birds directly. Thus, perceived positive impacts for wildlife can feedback into providing resources, without the need necessarily to experience wildlife (figure 1, feedback *c*).

Given the multiple potential negative impacts for wildlife of resource provisioning and that a concern for wildlife is clearly a significant motivating factor for people (e.g. [110]), it is interesting that so many people provide resources across such a broad range of species. This may represent a missing feedback loop, where people do not experience the negative impacts for wildlife, and so do not associate their actions with potential welfare issues (e.g. [38]). This may be a worrying symptom of a disconnect with the natural world. Negative social feedback and peer to peer dissemination of information to change behaviours is essential to raise awareness where provisioning is inappropriate [111]. Better management and education campaigns incorporating animal welfare into a framework to evaluate feeding activities may help people to recognize the harm that feeding often causes. Encouragingly, in the USA at least, although the numbers of animals observed in urban areas is reasonably stable (figure 2*d*) there appears to be a steadily decreasing trend in the number of people feeding wildlife other than birds around the home (figure 2*c*), suggesting that there may be cultural feedback towards recognizing potential problems associated with provisioning large animals.

## 7. In conclusion

Resource subsidies attract an increased abundance and often richness of species in close proximity to people, thereby enabling an increased frequency, duration and intensity of daily nature experiences. In the urban landscape, increased nature exposure across these three dimensions of dose has been positively associated with the health and wellbeing of people [12,13,15,96]. Ultimately, it is unclear to what degree variation in resource provisioning is driven by a desire to connect to nature, or that people who are connected to nature are more orientated towards providing resources. However, a strong sense of connection with nature is not a prerequisite for engaging in resource provisioning, so encouraging such activities, possibly through wildlife media, has the potential to reach those who are currently unengaged [103]. As urbanization continues, understanding how these areas can be best designed to foster people's desire to connect with everyday nature, while minimizing the potential negative impacts for wildlife, is of growing importance. For example, neighbourhoods with greater greenspace connectivity allow wildlife to move between gardens (and public greenspaces) more easily, thereby promoting interactions with a greater number of people [98]. Resource provisioning has the potential to contribute towards averting the extinction of experience, for the benefit of both people and wildlife.

## Data accessibility

This article has no additional data.
